# Comparative genomics of *Lupinus angustifolius* gene-rich regions: BAC library exploration, genetic mapping and cytogenetics

**DOI:** 10.1186/1471-2164-14-79

**Published:** 2013-02-05

**Authors:** Michał Książkiewicz, Katarzyna Wyrwa, Anna Szczepaniak, Sandra Rychel, Karolina Majcherkiewicz, Łucja Przysiecka, Wojciech Karlowski, Bogdan Wolko, Barbara Naganowska

**Affiliations:** 1 Institute of Plant Genetics, Polish Academy of Sciences, Strzeszyńska 34, 60-479, Poznań, Poland; 2 Laboratory of Computational Genomics, Institute of Molecular Biology and Biotechnology, Adam Mickiewicz University, Umultowska 89, 61-614, Poznań, Poland

**Keywords:** Narrow-leafed lupin, Glycine max, MFLP, Genome mapping, contigs, DNA sequencing, Synteny, BAC-FISH

## Abstract

**Background:**

The narrow-leafed lupin, *Lupinus angustifolius* L., is a grain legume species with a relatively compact genome. The species has 2n = 40 chromosomes and its genome size is 960 Mbp/1C. During the last decade, *L. angustifolius* genomic studies have achieved several milestones, such as molecular-marker development, linkage maps, and bacterial artificial chromosome (BAC) libraries. Here, these resources were integratively used to identify and sequence two gene-rich regions (GRRs) of the genome.

**Results:**

The genome was screened with a probe representing the sequence of a microsatellite fragment length polymorphism (MFLP) marker linked to Phomopsis stem blight resistance. BAC clones selected by hybridization were subjected to restriction fingerprinting and contig assembly, and 232 BAC-ends were sequenced and annotated. BAC fluorescence *in situ* hybridization (BAC-FISH) identified eight single-locus clones. Based on physical mapping, cytogenetic localization, and BAC-end annotation, five clones were chosen for sequencing. Within the sequences of clones that hybridized in FISH to a single-locus, two large GRRs were identified. The GRRs showed strong and conserved synteny to *Glycine max* duplicated genome regions, illustrated by both identical gene order and parallel orientation. In contrast, in the clones with dispersed FISH signals, more than one-third of sequences were transposable elements. Sequenced, single-locus clones were used to develop 12 genetic markers, increasing the number of *L. angustifolius* chromosomes linked to appropriate linkage groups by five pairs.

**Conclusions:**

In general, probes originating from MFLP sequences can assist genome screening and gene discovery. However, such probes are not useful for positional cloning, because they tend to hybridize to numerous loci. GRRs identified in *L. angustifolius* contained a low number of interspersed repeats and had a high level of synteny to the genome of the model legume *G. max*. Our results showed that not only was the gene nucleotide sequence conserved between soybean and lupin GRRs, but the order and orientation of particular genes in syntenic blocks was homologous, as well. These findings will be valuable to the forthcoming sequencing of the lupin genome.

## Background

Narrow-leafed lupin (*Lupinus angustifolius* L.) is a protein-rich grain crop for animal feed and human consumption that has excellent potential for sustainable crop rotation systems. The most important advantage of lupin cultivation is its contribution of fixed nitrogen and organic matter to soil, resulting in increased yields of successive crops [1].

During the last decade, several sets of molecular markers linked to various agronomic traits in narrow-leafed lupin have been developed using an innovative method, microsatellite-anchored fragment length polymorphism (MFLP) analysis [2]. In general, MFLP integrates amplified fragment length polymorphisms (AFLPs) [3] and simple sequence repeat (SSR)-anchor primer techniques [4]. MFLP gels generally have arbitrary patterns. However, a particular MFLP marker may be converted into a sequence-tagged site (STS) by excising a single band from the gel, cloning it into a vector, and sequencing the insert [2].

MFLP-derived STS markers have been generated for *L. angustifolius* genes tightly related to seed germination, flowering, and yield. Examples are *mollis,* which confers soft seediness (marker MoLi) [5]; *tardus* and *le*, which reduce pod shattering (markers TaLi, LeM1, and LeM2) [6,7]; and *Ku*, which removes the vernalization requirement (marker KuHM1) [8]. Furthermore, the MFLP method was used to develop sets of markers tagging hypothetical genes involved in lupin resistance to pathogenic fungi, including *Lanr1,* conferring anthracnose resistance (markers AntjM1 and AntjM2) [9-11]; *Phr1* and *Phr2*, carrying resistance to Phomopsis stem blight, a lupin disease caused by the necrotrophic fungus *Diaporthe toxica* (markers Ph258M1, Ph258M2, PhtjM1, and PhtjM2) [12,13]; Hua’an Yang, unpublished; and a pair of markers, RustM1 and RustM2, linked to rust-resistance loci [14].

Current advances in molecular biology have considerably accelerated progress on lupin genomics. First, a linkage map based on MFLP markers was constructed, and the regions carrying genes conferring valuable agronomic traits were localized [15]. This map provided general insight into lupin genomics, but the arbitrary nature of MFLP markers did not permit comparative mapping between narrow-leafed lupin and other legumes. Subsequently, a genetic map of a large set of gene-based PCR markers was drawn up. The use of sequence-specific markers enabled cross-species analyses, which resulted in the detection of conserved synteny between *L. angustifolius* and a legume model species, *Medicago truncatula*[16]. Consequently, these two maps were joined and supplemented with new molecular markers to create a reference genetic map of *L. angustifolius* aligned to the genome sequence of the model legume *Lotus japonicus*[17].

Comparative genomics studies between *L. angustifolius* and other species were not restricted to analyses of linkage maps, but also included DNA-hybridization methods. Screening of a narrow-leafed lupin cDNA library with *Glycine max* and *Arabidopsis thaliana* gene-derived probes showed a high level of gene structure conservation among these species [18]. In general, a syntenic network within conserved gene-rich regions of *G. max*, *M. truncatula*, and *A. thaliana* was discovered [19]. A further significant step was the completion of the *G. max* genome sequence [20]. The assembling and annotation of the *G. max*, *M. truncatula*, and *L. japonicus* genomes opened rich opportunities for translational genomics. A review of advanced genomic resources developed for legumes was reported by Sato et al. [21]. Most genes in papilionoid species occur within relatively large syntenic regions [22], facilitating cross-species gene annotation and positional cloning based on the sequenced genomes. However, the substantial genome rearrangements between *Medicago* and *Lotus,* which probably occurred during a polyploidy event, limited the syntenic conservation between the Genistoid and Millettioid clades [23] and has hampered comparative genomic studies between lupin and bean or pea.

The ability to screen the narrow-leafed lupin genome and discover genes was greatly enhanced by the development of new nuclear genome BAC libraries for the Polish cultivar ‘Sonet’ [24] and for the Australian cultivar ‘Tanjil’ [25]. The Sonet BAC library comprises 55,296 clones with an average insert size of 100 kb, representing approximately six haploid-genome equivalents. The Tanjil BAC library contains 111,360 clones with an average insert length of 99.7 kb, resulting in 12× coverage.

Recently, lupins have become the subject of extensive cytogenetic studies. Molecular cytogenetics have bridged the gap between the molecular and chromosomal levels of genome organization. Extended studies on genome size have been performed on both Old and New World species [26,27]. Further approaches have included chromosome structure analyses in *L. angustifolius* by FISH (fluorescence *in situ* hybridization) with different kinds of molecular probes [28] and PRINS (primed *in situ* labeling) [29]. The nuclear genome of *L. angustifolius* is partitioned into many small and morphologically similar chromosomes (2n = 40), so unambiguous chromosome identification without the use of molecular probes is not possible [28]. Critical for cytogenetic analysis was the construction of a nuclear genome BAC library for this species [24], because large insert libraries constitute unique resources for molecular probes in FISH-based studies. The BAC-FISH procedure proved valuable for physically mapping small plant genomes with numerous, tiny chromosomes, such as in *Phaseolus vulgaris*[30-32]. Furthermore, for *G. max*, whose whole-genome sequence is available, BAC-FISH with genetically anchored BACs allowed identification of all 20 chromosome pairs and their correlation with genetic and sequence-based markers [33]. In lupins, as in other plants without completely sequenced genomes, integrating gene sequences with physical localizations of BACs in chromosomes is of special importance. Few *L. angustifolius* linkage groups have been associated with appropriate chromosomes by the BAC-FISH procedure [34].

In the present study, we used an MFLP-derived marker as a probe to screen a narrow-leafed lupin BAC library. Because all publicly available molecular markers linked to *L. angustifolius* agronomic traits were developed by MFLP, we decided to verify whether such markers were useful for genome screening and, eventually, for positional cloning of particular genes. Complex molecular methods, such as DNA fingerprinting, BAC-FISH, and genetic mapping, were used to identify gene-rich regions of the lupin genome and to assign them to particular chromosomes and linkage groups. We exploited BAC-FISH to support contig construction, genetic mapping, and selection of repeat-free BAC clones for sequencing. Then, BAC sequences were functionally annotated and comparative mapping was performed to identify synteny blocks between *L. angustifolius* and *G. max* gene-rich genome segments.

## Results and discussion

### BAC library screening and construction of contigs

**Figure 1:**
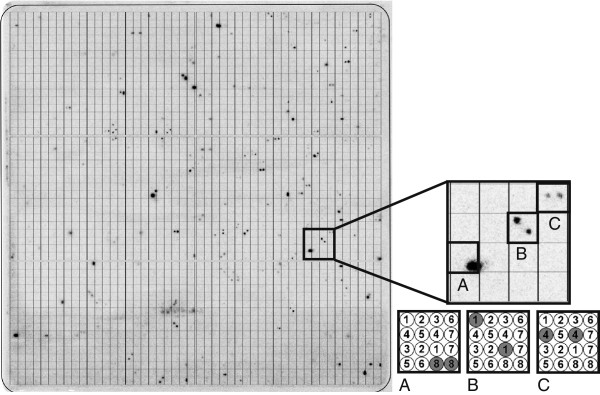
**Macroarray containing DNA of 18432 clones subjected to hybridization with PhtjM2 probe.** The enlarged fragment shows the arrangement of post-hybridization signals (**A, B, C**) and their coordinate system.

The hybridization probe was designed based on the MFLP-derived PhtjM2 marker sequence. Marker PhtjM2 is apparently linked with *Phr1*. RepeatMasker [35] analysis of the probe sequence [DDBJ:AB748564], with *A. thaliana* as a reference, revealed a short (27 nucleotides) section of simple repeats. A BLAST [36] search of the NCBI non-redundant (nr) DNA database [37] identified a region of 75–80 nucleotides (nt) within the probe with significant identity (75–80%) to numerous mouse and human sequences. Because of the central position of this non-specific region within the marker sequence, designing of a new probe without this section was impossible. The radioactively labeled probe was hybridized to a set of three macroarrays containing DNA of clones from the *L. angustifolius* nuclear genome BAC library. The screening procedure tagged 143 BAC clones (Figure 1). The positive hybridization signals were verified by PCR with DNA isolated from BAC clones as a template. Of the BAC clones, 137 generated only one PCR band with PhtjM2 primers, with no visible nonspecific amplification products on agarose gels. The remaining six BACs did not amplify and were recognized as false-positive hybridization signals.

Considering that the *L. angustifolius* BAC library has a 6× genome coverage, the number of BACs selected by the probe was equivalent to more than 20 copies in the genome. Therefore, MFLP-derived markers are not suitable for positional cloning. Did the BAC clones selected by the probe cluster in one locus within the genome or did they originate from various genome regions? To address this question, a restriction fingerprinting approach was used. Digestion of clones with two endonucleases (*Eco*130I and *Hin*dIII) yielded, on average, 30 products per clone. Products shorter than 200 nt were not counted. BAC clones that produced similar band patterns were considered to overlap and were grouped into contigs. From the 137 BACs, 19 contigs carrying 49 clones were assembled, while 88 BACs remained unlinked (singletons). The largest contig consists of five BAC clones.

### Functional annotation of BAC-end sequences

The 137 BACs selected by the PhtjM2 probe were subjected to BAC-end sequencing, generating 230 BAC-end sequences (BESs) with an average insert read length of 736 nt. Seventeen BAC clones could not be sequenced from either end, whereas sequencing failed from the 5^′^-end for six and from the 3^′^-end for four. BESs were deposited in DNA Data Bank of Japan (DDBJ) under consecutive accession numbers (from DDBJ:AB728840–AB729069).

During the RepeatMasker annotation, 38,983 nt (23.0% of the total BES length) were identified as transposable elements: 67.3% LTR/Copia and 23.2% LTR/Gypsy. Simple repeats accounted for 0.3% of the BES nucleotide collection. These proportions roughly corresponded to the recently published preliminary characterization of the narrow-leafed lupin genome [25]. The general percentage of repetitive content in BACs selected by the MFLP-based probe was higher than in the approach based on the annotation of 13,985 randomly selected BESs (23.3% versus 11.8%) [25]. In that preliminary lupin genomic survey, the most abundant transposable elements were LTR/Copia (52.0%) and LTR/Gypsy (34.5%). Considering these widespread repeats, the results of MFLP-based genome screening converged with those based on random BAC selection.

Next, repetitive sections of BESs were sequence-masked to identify hypothetical gene homologs. Fourteen gene-like sequences were identified. The presence of three genes (serine/threonine-protein kinase, glutamine synthetase, and H/ACA ribonucleoprotein complex subunit) was validated by statistically significant alignments with NCBI Expressed Sequence Tag (dbEST) [37] sequences from *Lupinus* spp., *Glycine* spp., *Lotus japonicus*, and *Medicago* spp. (see Additional file 1). Notably, the annotated genes have very diverse functions in plants and participate in a wide range of biochemical processes. Moreover, they may be expressed during physiological as well as pathological conditions. Such a result indicates that hybridization with the MFLP-derived probe picked up BAC clones representing dispersed sections of the *L. angustifolius* genome rather than a narrow set of clones linked with lupin resistance to Phomopsis stem blight.

### Identification of BAC clones giving single-locus FISH signals

Physical mapping of the BAC clones and functional annotation of the BESs allowed the selection of particular clones for cytogenetic analysis. The set of clones chosen for BAC-FISH contained: (i) all 11 clones carrying gene-like sequences in their BESs, (ii) 10 clones with no significant identity to any gene or repetitive sequence in both BESs, (iii) 11 clones carrying repetitive elements in one BES, and (iv) five clones containing repetitive elements in both BESs. BACs with various BES characteristics were chosen to verify whether BES sequence data could predict clone appearance in BAC-FISH (single or dispersed signals). Of the 37 BACs tested, 14 clones originated from 10 contigs, and 23 clones were singletons.

**Figure 2:**
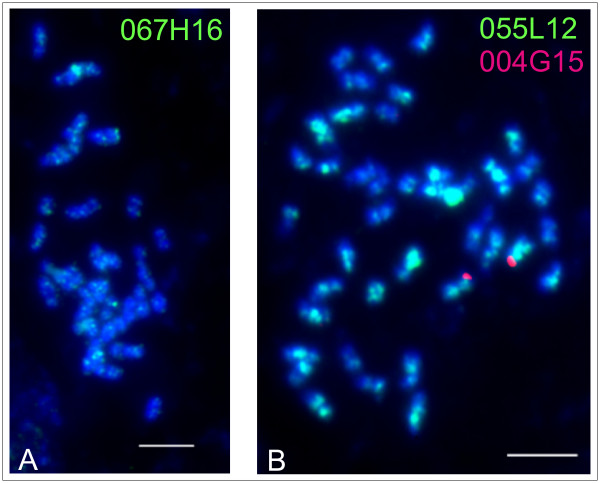
**Results of BAC-FISH analysis showing high vs low repetitive content within BAC clones. A**: BAC 067H16 (dispersed green signals) with 33.4% of repetitive content, **B**: BAC 055L12 (dispersed green signals) with 41% of repetitive content, and BAC 004G15 (single-locus red signals) with 7.5% of repetitive content. BAC clone DNA labeled with digoxigenin-11-dUPT (green signals) or tetramethylrhodamine-5-dUTP (red signals), chromosomes counterstained with DAPI. Scale bar = 5 μm.

BAC-FISH was performed to select BAC clones for full-insert sequencing. The BACs were used as molecular probes in the BAC-FISH procedure to directly visualize their chromosomal positions in the cytological preparation. This step of the analysis identified BAC clones with unique single-locus signals. Twenty-nine BACs were repetitive in BAC-FISH and eight had single-locus signals (Table 1). No correlation between the results of BAC-FISH (single-locus versus dispersed-loci) and the type of BES (gene-like or repetitive) was observed among the clones analyzed. The presence of transposable element(s) in BES(s) did not necessarily imply a repetitive appearance of a particular clone in BAC-FISH. Only a few of the analyzed BAC clones hybridized at one locus and were thus suitable for cytogenetic mapping. A large number of BACs gave FISH signals dispersed over many chromosomes, probably indicating the presence of repetitive sequences within their inserts. Such a pattern of hybridization has been reported in a number of other plant genome studies [38]. The results of BAC-FISH with two clones with dispersed signals, 055L12 and 067H16, are presented in Figure 2. Molecular cytogenetics offers some techniques to overcome such an obstacle, including hybridization with the C_0_*t* repetitive-DNA fraction or subcloning and selecting low-copy BAC clones. However, these approaches are not effective in all cases [30,31,39-41]. In the present experiment, to establish a collection of BACs suitable for subsequent multiBAC-FISH comparative analyses, it was important to select clones showing specific hybridization in the absence of any reagent directed against repetitive content.

**Table 1:** List of BAC clones analyzed in BAC-FISH and their assignment to linkage groups

**BAC clone**	**Contig**	**BAC-FISH**	**Genetic markers, accession**	**Linkage group**	**BAC-end annotation**	
					**3**^**′**^**end**	**5**^**′**^**end**
003M07	-	repetitive	-	-	Ty1/Copia	Ty1/Copia
004G15	-	**single locus**	**004G15_3** [Genbank:GF111994]	**5**	gene	-
008K19	-	repetitive	-	-	-	-
009I22	-	repetitive	-	-	gene	(TA)n, (TAA)n
019P14	3	repetitive	-	-	-	-
024B01	-	repetitive	-	-	Ty1/Copia	-
032A19	7	repetitive	-	-	gene	gene
032O04	9	repetitive	-	**-**	Ty1/Copia	-
036J18	12	repetitive	-	-	Ty1/Copia	Ty1/Copia
044M22	3	repetitive	-	-	-	Ty1/Copia
048B06	-	repetitive	-	-	Ty3/Gypsy	Ty3/Gypsy
050C12	8	repetitive	-	-	gene, Ty1/Copia	Ty3/Gypsy
055L12	5	repetitive	**055L12_13** [Genbank:GF111997]	**6**	Ty1/Copia	-
057J20	-	**single locus**	**057J20_5** [Genbank:GF111995]	**3**	-	-
057K22	-	**single locus**	**057K22_3F2** [Genbank:GF112002]	10*	-	-
058D20	-	repetitive	-	**-**	-	-
063F11	-	repetitive	-	-	-	-
064L14	5	repetitive	-	-	gene	-
065K14	-	repetitive	-	-	-	-
067H16	-	repetitive	**067H16_41** [Genbank:GF111998]**, 067H16_43** [Genbank:GF111999]	**6**	Ty1/Copia	-
074I10	-	**single locus**	**074I10_3_1** [Genbank:GF112001]**, 074I10_3_2** [Genbank:GF112000]	3, 7*	Ty1/Copia	Ty3/Gypsy, (TA)n
075A18	-	repetitive	-	-	Ty1/Copia	Ty1/Copia
076K16	4	**single locus**	**076K16_3F3** [Genbank:1243111]**, 076K16_5** [Genbank:GF112014]	6*	-	gene
077C13	-	**single locus**	**077C13_3** [Genbank:GF112003]	10*	-	Ty1/Copia
077C14	4	repetitive	-	-	-	-
078H13	18	repetitive	-	-	-	Ty3/Gypsy
079I08	1	repetitive	-	-	Ty1/Copia	-
080B11	-	**single locus**	**080B11_3** [Genbank:GF111996]	6*	-	-
083C06	12	**single locus**	-	20*	gene	Ty1/Copia
083N13	13	repetitive	-	-	gene	-
085B02	-	repetitive	-	-	gene	gene
086G16	-	repetitive	-	-	-	-
088J02	-	repetitive	-	-	Ty1/Copia	gene
095H17	-	repetitive	-	-	-	MuDR
095I10	-	repetitive	-	-	RC/Helitron	gene
096J11	-	repetitive	-	-	-	-
096K01	-	repetitive	-	-	-	Ty3/Gypsy

* linkage group assignment confirmed by BAC-FISH with two clones.

### BAC clone sequencing and annotation

Based on the results of contig construction, BESs annotation, and preliminary BAC-FISH experiments, five clones were chosen for complete 454-type sequencing. This set included two clones with BAC-FISH signals dispersed over numerous chromosomes: 055L12, representing the largest contig, and 067H16, classified as a singleton. Clones behaving as repetitive in FISH were assumed to contain a big fraction of transposable elements, whereas clones with unique cytogenetic signals were expected to possess more gene sequences. Based on these assumptions, three single-locus BACs were sequenced: 004G15, 057J20, and 080B11. Sequencing of all five BACs resulted in the assembling of two contigs for the 055L12 clone (96,519 nt), four for 067H16 (92,686 nt), two for 004G15 (86,908 nt), four for 057J20 (87,213 nt), and two for 080B11 (88,445 nt). Full BAC sequences were assembled by joining contigs together. Gaps were indicated by separators of 100 ambiguous nucleotides.

As a result of RepeatMasker analysis (Table 2), 41.0% of the 055L12 and 33.4% of the 067H16 BAC sequences were determined to carry repetitive content. The main groups of repeats were retrotransposons representing Ty1/Copia for 055L12 and Ty3/Gypsy for 067H16. Clones with single-locus BAC-FISH signals contained a considerably lower percentage of repetitive content in their sequences. The fraction of interspersed repeats comprised 7.5% of BAC clone 004G15 and 18.0% of 057J20. Within the sequence of 080B11, no transposable element was identified.

**Table 2:** Repetitive elements identified by RepeatMasker in the sequences of BAC clones

**Repetitive content**	**Percentage share**				
	**055L12[EMBL:HE804809]**	**067H16[EMBL:HE804811]**	**004G15[EMBL:HE804808]**	**057J20[EMBL:HE804810]**	**080B11[EMBL:HE804812]**
**Retrotransposons**	**38.2%**	**32.7%**	**6.9%**	**17.3%**	**-**
LINE	0.8%	-	0.5%	6.8%	-
Ty1/Copia	30.3%	6.0%	-	4.8%	-
Ty3/Gypsy	7.0%	26.7%	6.4%	5.8%	-
**Transposons**	**2.8%**	**0.6%**	**0.1%**	**0.4%**	**-**
En-Spm	2.3%	-	-	-	-
MuDR	0.5%	-	0.1%	0.0%	-
hobo-Activator	-	0.6%	-	-	-
RC/Helitron	-	-	-	0.4%	-
**Unclassified**	**-**	**0.1%**	**0.5%**	**0.3%**	**-**
**Total interspersed repeats**	**41.0%**	**33.4%**	**7.5%**	**18.0%**	**-**
Simple repeats:	0.1%	0.2%	0.1%	-	0.1%

**Figure 3:**
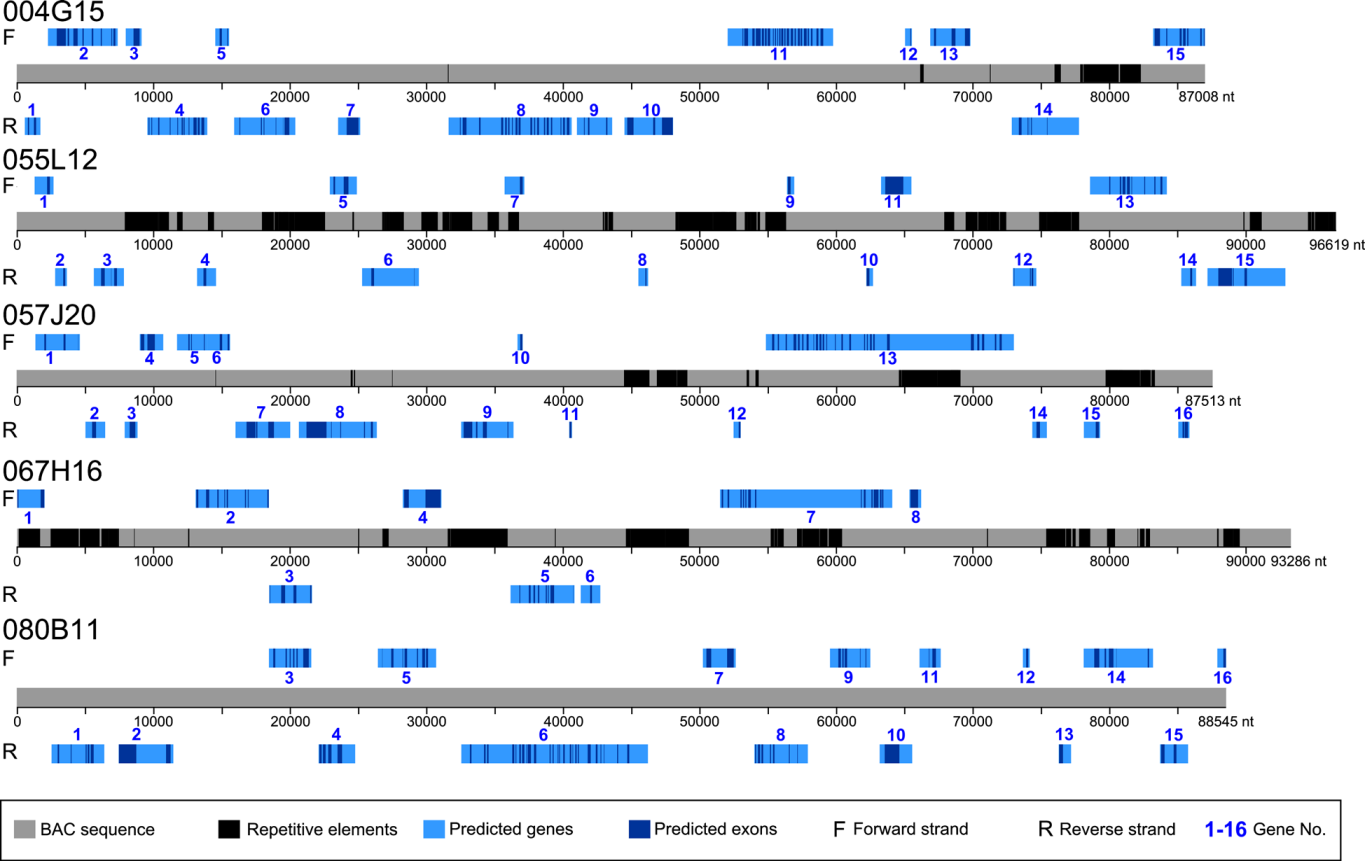
**Genes identified by FGENESH prediction in sequenced *L. angustifolius *BAC clones: 004G15, 055L12, 057J20, 067H16 and 080B11.** Predicted genes are marked with numbers 1–16 corresponding to annotation data shown in Table 3.

BAC sequences with masked repetitive regions were subjected to FGENESH [42,43]*in silico* gene prediction followed by BLAST against the EST, nr, and Swiss-Prot [44] collections (Table 3). In the structures of 055L12 and 067H16, the BACs containing the highest ratios of repetitive content, several gene and gene-like sequences were identified. The most significant similarities were to glucan endo-1,3-beta-glucosidase 5, a GRAS-family transcription factor, a sialyltransferase-like protein, an auxin-induced protein 6B, and endonuclease V. In particular, for the BAC 055L12, which was the richest in transposable sequences, 87.7% of FGENESH predictions had no coverage in the EST collection. Within the three single-locus BAC clone sequences, large gene-rich regions (GRRs) were identified. The majority of genes predicted by FGENESH in these BACs (32 of 47, or 68%) were validated by statistically significant similarities to sequences from the EST and NCBI protein nr collections. For 13 predicted genes (approximately 28%), the sequence equivalents were also recognized in Swiss-Prot database (with e-value cut-off 1e-50). The schematic structures of the sequenced BAC clones, showing repetitive elements and predicted genes, are presented in Figure 3. BAC sequences with annotations were deposited in the European Molecular Biology Laboratory (EMBL) Nucleotide Sequence Database under accession numbers 004G15 [EMBL:HE804808], 055L12 [EMBL:HE804809], 057J20 [EMBL:HE804810], 067H16 [EMBL:HE804811], and 080B11 [EMBL:HE804812].

**Table 3:** List of genes predicted in BAC clones and confirmed by annotation to Swiss-Prot, nr and EST collections with e-value cut-off 1e-50

**FGENESH predictions**			**Alignment e-values**		
**BAC clone, accession**	**Gene no.**	**Annotations by sequence similarity**	**est_other**	**nr**	**Swiss-Prot**
004G15, [EMBL:HE804808]	1	iron-sulfur assembly protein IscA-like 1	2.0E-54	1.9E-49	7.0E-21
	2	cell division protein ftsZ homolog 2-1	0	0	0
	3	unknown gene	1.7E-63	2.7E-62	-
	4	probable cytosolic iron-sulfur assembly protein	0	0	1.7E-110
	5	cytochrome b-c1 complex subunit 9-like	3.4E-75	2.2E-53	2.4E-30
	6	iron-sulfur assembly protein IscA-like	1.3E-67	6.5E-60	1.4E-29
	7	unknown gene	6.0E-105	2.5E-98	-
	8	signal recognition particle 54 kDa protein	0	0	0
	9	small nuclear ribonucleoprotein G-like	1.1E-69	1.4E-69	7.6E-32
	10	unknown gene	4.1E-103	0	-
	11	125 kDa kinesin-related protein-like	2.3E-180	0	0
	13	domain-containing protein 90-like	0	9.4E-173	2.1E-70
	14	vacuolar protein sorting-associated protein 55	3.9E-147	6.1E-146	7.2E-38
	15	N-acetylglucosaminyltransferase SPINDLY-like	0	0	3.9E-177
055L12, [EMBL:HE804809]	11	GRAS family transcription factor	0	0	2.3E-170
	13	aldehyde dehydrogenase family 3 member F1	3.2E-77	4.4E-64	4.1E-20
057J20, [EMBL:HE804810]	6	unknown gene	8.6E-78	7.3E-80	-
	7	formimidoyltransferase-cyclodeaminase-like	1.7E-176	2.2E-176	1.0E-06
	8	probable galacturonosyltransferase 10	0	0	0
	9	protein TIC 20-IV, chloroplastic, precursor	1.4E-164	0	1.1E-64
	13	vacuolar protein sorting-associated protein 18	0	0	2.3E-155
	14	unknown gene	6.4E-60	1.0E-26	-
	16	unknown gene	3.2E-53	4.9E-52	-
067H16, [EMBL:HE804811]	2	unknown gene	2.4E-116	8.7E-117	-
	3	unknown gene	0	8.1E-160	-
	4	glucan endo-1,3-beta-glucosidase 5-like	0	0	0
	5	endonuclease V	1.9E-117	5.3E-113	3.5E-67
	7	sialyltransferase-like protein	0	0	3.8E-12
	8	auxin-induced protein 6B	1.9E-121	1.4E-130	1.1E-17
080B11, [EMBL:HE804812]	1	50S ribosomal protein L35	7.2E-121	7.9E-90	-
	2	unknown gene	0	0	-
	3	myb family transcription factor APL-like	7.6E-167	0	3.5E-43
	4	isoflavone reductase homolog	0	0	0
	5	unknown gene	6.2E-111	7.9E-111	-
	6	unknown gene	0	0	-
	7	transmembrane protein 45B	0	0	2.4E-08
	8	MOB kinase activator-like 1	0	0	1.1E-148
	9	unknown gene	2.2E-51	6.6E-53	-
	10	histone-lysine N-methyltransferase ASHR2-like	5.7E-119	1.7E-177	2.5E-103
	14	protein BREVIS RADIX-like	0	0	1.3E-129

### Microsynteny between *L. angustifolius* and *Glycine max*

**Figure 4:**
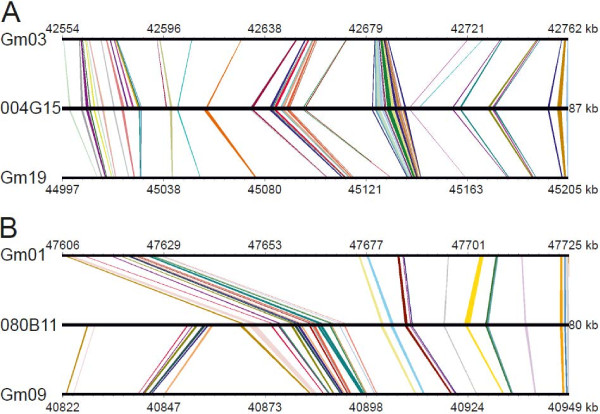
**Synteny between gene-rich regions of *L. angustifolius *and *Glycine max. ***004G15 (**A**) and 080B11 (**B**): narrow-leafed lupin BAC clones mapped in linkage groups NLL-05 and NLL-06. Gm01, Gm03, Gm09, Gm19: soybean chromosomes. The order and orientation of syntenic blocks is visualized by colored homology links.

Two large repeat-free gene-rich regions from BAC clones 004G15 and 080B11 served as anchors for comparative DNA sequence analysis between the genomes of *L. angustifolius* and *G. max*. Strong and conserved synteny to *G. max* was identified for both GRRs (Figure 4). The sequence of BAC clone 004G15 had two strikingly similar regions on soybean chromosomes Gm03 and Gm19. The high level of sequence conservation was illustrated both by the identical order of microsyntenic blocks in soybean chromosomes and by their concerted orientation. No syntenic sequence was reversed in this region, indicating that these two segments of the *G. max* genome were not involved in chromosomal rearrangements that changed the gene order. However, those sections of soybean chromosome were approximately twice as big as their narrow-leafed lupin equivalents.

Sequences homologous to *L. angustifolius* GRR 080B11 were identified in soybean chromosomes Gm01 and Gm09. No evidence of a rearrangement or reorientation was found. The organizational parallels to the soybean chromosomes were not as precise as with clone 004G15. Two regions of hypothetical insertions in *G. max* chromosomes were localized around the 47,650 kb locus in Gm01 and the 40,860 kb locus in Gm09. Because these loci are not homologous, the insertions must have occurred after the duplication of the soybean genome.

The genome of *Glycine* is characterized by a relatively recent duplication event that took place about 13 Mya. Much of the DNA generated in that process should remain in the soybean genome. This implies that a given region of the *Medicago* or *Lotus* genome should correspond to two regions in *Glycine*[22]. Our comparison of two *Lupinus* whole BAC clone sequences with *Glycine* sequences showed the high extent of microsynteny between them. Each lupin clone fit surprisingly well two soybean sequences, with no inversions evident.

Comparative genome analyses and the possibility of information transfer among species depend on their phylogenetic distances. *Lupinus angustifolius* and the three legume models for which genome sequence information is available – *G. max*, *M. truncatula* and *L. japonicus* – belong to different phylogenetic clades. Thus, none of the model species is closely related to *L. angustifolius*. We chose *G. max* as a reference model because it has the same number of small, morphologically-similar chromosomes (2n = 40). Furthermore, Gao et al. [25] sequenced both ends of 9,600 randomly selected BAC clones from their *L. angustifolius* library and generated 13,985 BESs, covering approximately 1% of the genome. They found that the average percent identity of BLASTn alignments between *L. angustifolius* BESs and *G. max*, *M. truncatula,* and *L. japonicus* was 95.8%, 94.4%, and 90.6%, respectively. Our results indicated that not only were the nucleotide sequences of genes conserved between the soybean and lupin genomes, but also that the order and orientation of genes in syntenic blocks were maintained.

### Genetic mapping of selected BAC clones

All clones showing single-locus BAC-FISH signals were genetically mapped. To address this task, PCR primers were designed on the basis of BESs originating from particular BACs. PCR products amplified from DNA isolated from parental lines of the mapping population were polymorphic in the following six BESs: 004G15_3, 057J20_5, 080B11_3, 074I10_3, 077C13_3, and 076K16_5. Notably, the BES 074I10_3 had two polymorphic PCR products, both exposing a dominant type of segregation. Polymorphism was observed neither for amplicons tagging both ends of BAC clones 057K22 and 083C06 nor for PCR products derived from BESs 004G15_5, 057J20_3, 080B11_5, 074I10_5, 077C13_5, and 076K16_3. Considering the quality of BESs and the monomorphic nature of particular PCR products, new primers were designed to elongate the BESs by Sanger sequencing. After the first cycle of BES elongation, polymorphism was detected for the 3^′^ BES of clone 057K22, which was renamed 057K22_3F2. The second round of BES extension identified a polymorphic site in the sequence of 076K16_3F3. No polymorphism was detected in products derived from BAC clone 083C06, despite four cycles of BES elongation, primer design, and subsequent PCR product sequencing.

Additionally, primers were designed for selected “454” contigs of BAC clones 055L12 and 067H16 to incorporate these sequences into the *L. angustifolius* genetic map. For clone 055L12, PCR product polymorphism was observed in contig 1 (055L12_13). Two polymorphic amplicons were identified for contig 4 of BAC clone 067H16 (067H16_41 and 067H16_43).

In the present work, 12 new genetic markers originating from nine BAC clones and differing in the method of polymorphism detection were developed. Six markers can be simply scored by PCR and agarose gel electrophoresis, because they segregated as present/absent or because the PCR products differed considerably in size. Four markers required endonuclease digestion of the amplicons (Cleaved Amplified Polymorphic Sequence, CAPS) [45], whereas for two markers, the restriction stage had to be preceded by PCR amplification with primers that introduced a restriction site based on a polymorphic locus (derived CAPS, dCAPS) [46]. Sequences of the PCR products from the parental lines of the mapping population were compared with the source BESs and BAC sequences. The identity levels of at least 98% were observed for all but one marker, 074I10_3_2 designed for BES 074I10_3. However, the primer pair based on the sequence of 074I10_3 amplified two polymorphic PCR products, and the other, 074I10_3_1, showed 98.4% nucleotide identity with 074I10_3. Considering the similarity of marker sequences to reference BESs and BACs, all markers except 074I10_3_2 were considered to be specific labels of appropriate BAC clones.

Molecular markers were allocated to five linkage groups of the recently published narrow-leafed lupin genetic map [17]. Two markers, 067H16_41 and 067H16_43, derived from two regions of BAC clone 067H16, were positioned in linkage group NLL-06 at a reciprocal distance of 0.5 cM. Because the source sequences are physically linked, close genetic localization of derivative markers was expected. Two other markers for sequenced BACs mapped to this chromosome: 055L12_13, tagging a contig of five clones, and 080B11_3, representing a singleton. Merely 10 cM away from 080B11_3, a pair of markers (076K16_3F3 and 076K16_5) originating from BAC clone 076K16 was also localized. Two other markers, 077C13_3 and 057K22_3F2, based on singletons, were placed in the group NLL-10 within an interval of 1.2 cM. Two genetic markers, 074I10_3_1 and 074I10_3_2, derived from the same BES, mapped to the linkage groups NLL-07 and NLL-03, respectively. As mentioned above, comparative sequence analysis of these two markers and BES 074I10_3 suggested that only 074I10_3_1 reflected the position of BAC clone 074I10. Genetic markers 004G15_3 and 057J20_5, derived from the two remaining sequenced BACs, were localized in linkage groups NLL-05 and NLL-03, respectively. A supplementary file contains detailed marker data including primer sequences, PCR product sizes, sequence identity levels, applied detection methods, linkage group assignment, LOD scores, and segregation data for *L. angustifolius* 83A:476 (D) and P27255 (W) recombinant inbred lines [see Additional file 2.

### BAC-FISH as a supporting tool in genetic mapping

A comprehensive cytogenetic approach was applied to support the linkage mapping of selected BAC clones. The co-localization of different clones was analyzed using the multiBAC-FISH variant, i.e., simultaneous application of several differently labeled BACs on the same slide. The power of the FISH technique is limited by two parameters: probe-size detection and axial-resolution. In advanced FISH variants (e.g., on extended fiber preparations) both parameters are high, and even gene-sized DNA fragments can be mapped [47]. The sensitivity and resolution of FISH in mitotic metaphase are relatively low [48]. Nevertheless, our study was based on observations of metaphase mitotic chromosomes, because we needed to analyze the clone positions with reference to individual chromosomes and linkage groups. This strategy involved all recognized PhtjM2 single-locus BACs. Additionally, one supplementary BAC clone, 017B07, that previously mapped to linkage group NLL-20 [Michał Książkiewicz, unpublished] was included in this analysis. The specifications of the BAC-FISH combinations are presented in Table 4.

**Table 4:** Localization of cytogenetic markers in narrow-leafed lupin chromosomes: BAC pairs tested in complex BAC-FISH analysis

**Linkage group**	**BAC**									
**5**	**004G15**	**004G15**								
**3**	**057J20**	-	**057J20**							
**10**	**057K22**	no	no	**057K22**						
**3, 7**	**074I10**	no	no	-	**074I10**					
**-**	**076K16**	no	no	-	-	**076K16**				
**10**	**077C13**	no	no	**yes**	no	no	**077C13**			
**6**	**080B11**	-	-	no	no	**yes**	no	**080B11**		
**-**	**083C06**	no	no	-	-	-	-	no	**083C06**	
**20**	**017B07**	**-**	-	no	no	no	no	-	**yes**	**017B07**

no: BAC-FISH signals for clones from this pair were observed in different chromosomes.

yes: BAC-FISH signals for clones from this pair were observed in the same chromosome.

The BAC-FISH signals served to determine clone location in chromosomes as well as to elaborate and verify their linkage group assignments. Cytogenetic analyses were also used to validate the physical linkage of clones that were initially assembled into contigs by the restriction fingerprinting approach. BAC-FISH proved to be helpful in checking individual contig positions within the lupin genome. Signals of BAC clones (076K16 and 083C06) originating from two contigs (4 and 12) detected on different chromosomes confirmed that these contigs were separate. The location of BACs representing the same contig in different chromosomes proved that the contig did not reflect the real structure of the genome region, and a correction of bioinformatics analyses was necessary. Such an outcome was observed for BACs 057J20 and 074I10 and resulted in the classification of these clones as singletons.

BAC FISH was also indispensable during linkage mapping. BAC-FISH with clones 074I10 and 057J20 helped to resolve the issue of double genetic markers for BAC clone 074I10, which mapped to two different linkage groups. The preliminary assumption that the marker 074I10_3_2 was not physically linked to a particular BAC clone proved correct, because the FISH signals specific for BACs 074I10 and 057J20 were observed on separate chromosomes. Because marker 057J20_5 mapped clone 057J20 to linkage group NLL-03, the fact that these two BACs were not co-localized indicated that BAC 074I10 was not located in NLL-03. For the remaining BACs, every pair of clones that was not co-localized by genetic mapping had BAC-FISH signals on separate chromosomes.

Two clones, 057K22 and 077C13, were localized in the same locus. Based on the resolution of BAC-FISH on metaphase chromosomes, these sequences cannot be separated by more than a few megabases. This result converges with the linkage mapping outcome, because the markers 057K22_3F2 and 077C13_3 were both located in linkage group NLL-10 at a distance of 1.2 cM. Similarly, clones 080B11 and 076K16 mapped to NLL-06. Their BAC-FISH signals overlapped each other in one chromosome, but these BACs were separated by a genetic distance of 10 cM.

**Figure 5:**
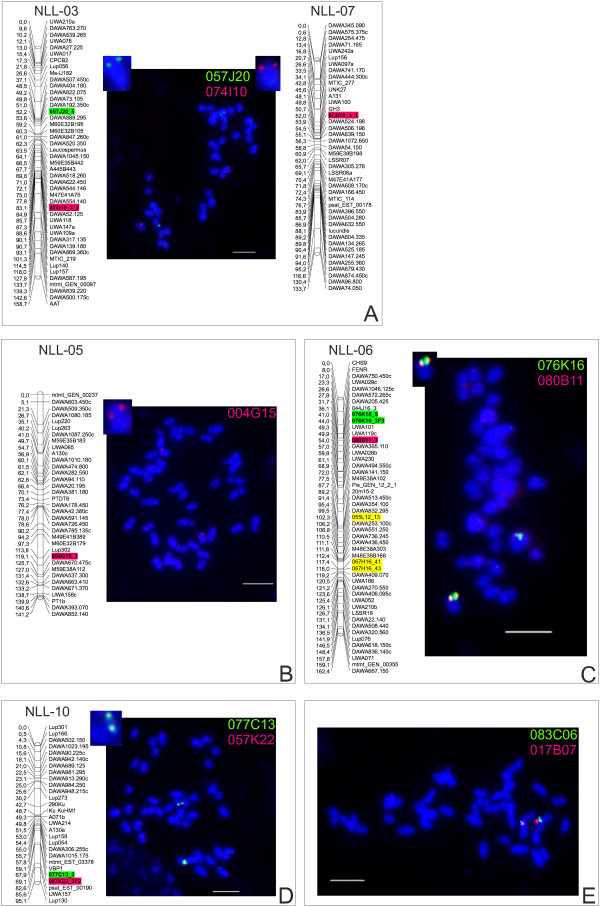
**Integration of *L. angustifolius *genetic and physical maps: assignment of linkage groups (NLLs) to corresponding chromosomes by BAC-FISH. A:** Localization of two BAC clones 074I10 (red signals) and 055L12 (green signals) in two different chromosomes. BAC 074I10 position is represented by genetic marker 074I10_3_1, mapped in NLL-07 (see Results and Discussion). **B:** Genetic marker 004G15_3 (generated from BAC 004G15, red signals) mapped in NLL-05. **C:** Three genetic markers (076K16_5, 076K16_3F3 and 080B11_3), generated from BAC clones 076K16 (green signals) and 080B11 (red signals), mapped in NLL-06 and co-localized in the same chromosome site (overlapping signals). Yellow colored genetic markers on NLL-06 graph gave dispersed BAC-FISH signals and are not shown in this figure. **D:** Two genetic markers 077C13_3 (green) and 057K22_3F2 (red) mapped in NLL-10 and corresponding BACs co-localized in the same chromosome site (yellow signals). **E:** BAC 083C06 (green signals) co-localized in the same chromosome arm as BAC 017B07 (red signals) previously mapped in linkage group NLL-20. BAC clone DNA labeled with tetramethylrhodamine-5-dUTP (red signals) and digoxigenin-11-dUPT (green signals), chromosomes counterstained with DAPI. Overlapping signals - yellow. Scale bar = 5 μm.

As mentioned above, the BAC clone 083C06 remained unmapped, despite four rounds of BAC-end sequence elongation and PCR-product analysis. To address its chromosome localization, this clone was used in BAC-FISH together with clone 017B07, which mapped to linkage group NLL-20. Hybridization signals for these two clones were observed on the same chromosome arm, in two close but separate loci. Given the FISH resolution and approximate ratio of physical to genetic distance, such a result indicates a genetic linkage of clones 083C06 and 017B07 of more than 10 cM. The linkage groups, supplemented with newly designed genetic markers and assigned chromosomes carrying specific BAC-FISH signals, are presented at Figure 5.

The first three genetic linkage groups of *L. angustifolius* were assigned to chromosomes using a similar approach, based on hybridization of SSR-rich probes with the BAC library, selection of single-locus clones in BAC-FISH, and subsequent mapping of these clones using BES markers [34]. Here, we significantly enhanced the integration of the chromosomal and genetic maps of narrow-leafed lupin by associating another five linkage groups with chromosomes. Because 40% of the lupin karyotype has now been assigned to the genetic map, an alternate strategy can be used to complete the integration process. A commonly used approach to build an integrated plant genome map is FISH of BAC clones selected from the library by probes carrying sequences of genetically mapped markers [30,31]. However, the markers must be carefully selected to avoid incorporating repetitive sequences and duplicated genes, which can produce an excessive number of hybridization signals. The most recent genetic map of narrow-leafed lupin contains more than 190 gene-based markers [17]. A subset of these markers originates from putatively single- or low-copy genes and may be used in the future for a marker-oriented integration of the linkage and cytogenetic maps of this species.

## Conclusions

Probes based on sequences of MFLP-derived markers are not suitable for positional cloning of genes linked to the particular markers, because they hybridize to numerous loci throughout a genome. However, such probes can be useful in general screening of a genome, because both the amount of repetitive DNA and the proportions between the main groups of transposable elements in BAC clones selected by MFLP probe are similar to those in randomly chosen BACs.

BAC-FISH can serve as a supporting tool to select BAC clones for sequencing and tagging gene-rich regions in species with low quantities of sequence data. BAC clones showing FISH signals dispersed across numerous chromosomes contained many transposable elements, whereas in clones hybridizing to single loci, the amount of repetitive DNA was negligible. Moreover, single-locus BACs carried significantly more gene-like sequences than clones tagged by FISH as repetitive.

Gene-rich regions identified in the *L. angustifolius* genome contain a low number of interspersed repeats and a high degree of synteny with the genome of the model legume *G. max*. This information is valuable for the lupin genome sequencing project. Such sections of the genome can be readily assembled to large scaffolds and their gene content properly annotated.

## Methods

### BAC library screening

The sequence of MFLP-derived marker, PhtjM2, apparently linked with *Phr1* gene conferring resistance to Phomopsis stem blight, was used for BAC library screening [12,13, Hua’an Yang - unpublished]. The PCR product [DDBJ:AB748564] was amplified with primers PhtjM2F: TTTGTAGATGTTTTCTTTCC and PhtjM2R: CCAAGCATTTATGTTCTACC using *L. angustifolius* genomic DNA as a template. The PCR product was purified (QIAquick PCR Purification Kit, Qiagen) and radiolabeled by random priming (HexaLabel DNA Labeling Kit, Fermentas) with 50 μCi [α-32P]-dCTP. Additionaly, the PCR product was sequenced on the ABI PRISM 3130 XL Genetic Analyzer (Applied Biosystems, Hitachi) with the PhtjM2F and PhtjM2R primers. The probe sequence was checked in RepeatMasker for the presence of repetitive content.

The nuclear genome BAC library of *L. angustifolius* cv. Sonet was used for screening [24]. High-density macroarrays carrying DNA isolated from BAC clones were prepared with a GeneTAC G3 robotic station (Genomic Solutions) on Hybond N^+^ 22.2 × 22.2 cm nylon filters (AP Biotech, Little Chalfont, UK). BAC clones were spotted on macroarrays in two copies, organized in specific coordinates, to enable distinction between true results and false positive hybridization signals. The whole BAC library was represented by a set of three blots.

Hybridization of the probe with DNA macroarrays was carried out for 16 h at 60°C in a HYBSOL, composed of: 5× SSC (0.75 M NaCl, 0.075 M sodium citrate), 5× Denhardt’s Solution (0.1% w/v Ficoll-400, 0.1% w/v polyvinylpyrrolidone, 0.1% w/v BSA), 0,5% w/v SDS. Post-hybridization washes at 60°C were performed successively (5× SSC and 0.5% SDS for 1 min, 5× SSC and 0.5% SDS for 20 min, 2.5× SSC and 0.25% SDS for 20 min, 1.25× SSC and 0.1% SDS for 20 min, 0.5× SSC and 0.05% SDS for 20 min). Afterwards, macroarrays were exposed for 24–48 h to BAS-MS 2340 imaging plates (Fujifilm) and scanned using a FLA-5100 phosphoimager (Fujifilm). BAC clones showing positive hybridization signals were subjected to DNA isolation procedure (BAC DNA Kit, Sigma). Verification of BAC clones was performed by PCR with the PhtjM2F and PhtjM2R primers.

### PCR and electrophoresis conditions

Primers for PCR were designed in Primer3Plus [49]. The amplification reactions were set up in 96-well twin.tec PCR plates (Eppendorf) using 0.5 U Taq DNA Polymerase Recombinant (Invitrogen) supplied with 1× PCR buffer and 2 mM Mg^2+^, 0.25 mM dNTP, 0.25 μM each primer, 25 ng DNA template and deionized water up to 20 μl. The amplification procedure involved initial denaturation (94°C, 4 min), then 35 cycles consisting of three steps: annealing (45-62°C, 30 s), elongation (72°C, 40 s) and denaturation (94°C, 30 s), followed by final elongation (72°C, 6 min). The PCR products were visualized by electrophoresis on 1% agarose gel (2 h, 6 V/cm, 21°C) and ethidium bromide staining. Orange DNA Loading Dye (Fermentas) was used for loading samples on agarose gel whereas O’GeneRuler 1 kb Plus DNA Ladder (Fermentas) was applied for sizing and quantification of DNA fragments.

### BAC-end sequencing and BAC clone sequencing

BAC-ends were sequenced on the ABI PRISM 3130 XL Genetic Analyzer (Applied Biosystems, Hitachi) using pIndigoBAC5 sequencing primers:

3^′^- GGATGTGCTGCAAGGCGATTAAGTTGG

5^′^- CTCGTATGTTGTGTGGAATTGTGAGC

Chromatograms were verified in Chromas Lite 2.01 for base-calling errors and exported as BAC-end sequences (BES) in the FASTA format. BESs obtained with the use of the 3^′^ primer were named with “_3” at the end, and 5^′^ primer BESs with “_5”.

BAC clones selected for the whole insert GS FLX TITANIUM 454 DNA Sequencing were delivered to LGC Genomics (Germany). Sequencing was performed with tagged BAC DNA samples using 1/8 picotiterplate (PTP). Considering even distribution of reads, the planned sequencing scheme was equal to approximately 10× coverage of 454 reads for each BAC clone. Sequences were assembled by LGC Genomics.

### Functional annotation of BAC-end and BAC sequences

Precise characteristics of various genetic elements encoded in the BES sequences were revealed by *in silico* annotation. The process of annotation included *de novo* detection of specific signals located on the genomic sequence as well as comparative analysis. The procedure was executed with an analysis pipeline specifically designed for gene discovery and comparative genome research (Karlowski unpublished). Repetitive elements were identified using sequences deposited in the RepBase, TIGR and MIPS Plant Repeats Collections [50,51]. The following cut-off e-values were applied for constructed alignments: 1e-11 for transposable DNA and 1e-10 for proteins. BAC-end sequences containing recognized repetitive segments were subsequently masked, whereas the remaining BESs from the collection were subjected to BLAST comparative analysis with DNA and protein sequences from the EMBL Nucleotide Sequence Database, GenBank, DNA Database of Japan, RCSB Protein Data Bank, Swiss-Prot, Protein Information Resource and Protein Research Foundation [37,44], with an e-value cut-off of 1e-10 [52]. Additionally, EST sequences of *Lupinus* spp., *Lotus japonicus*, *Medicago* spp. and *Glycine* spp*.* were incorporated to the analysis.

The first step of BAC sequence annotation was to identify repetitive content in DNA based RepeatMasker [35] with the reference to *Arabidopsis thaliana*. Masked sequences were analyzed in protein based RepeatMasker to identify elements not present in the *A. thaliana* DNA repeats database. BAC sequences with masked repetitive content were subjected to FGENESH [42,43] gene recognition with the *M. truncatula* genome as a reference. The sequences of translated proteins were searched for similarity against Swiss-Prot, nr protein collections and EST accessions.

### Microsynteny analysis

BAC sequences, with repetitive content and low complexity regions masked, were used for sequence homology search against the *Glycine max* genome. Sequence similarity analysis was performed in CoGe BLAST [53]. The following parameters were set: e-value cut-off 1e-20, word size 8, gap existence cost 5, gap elongation cost 2, nucleotide match score 1, nucleotide mismatch score −2. The visualization of syntenic blocks was done in Web-based Genome Synteny Viewer [54].

### Restriction fingerprinting and contig assembly

The BAC DNA was digested with *Eco*130I and *Hin*dIII enzymes in separate reactions. Two units of the enzyme were applied for 1 μg of BAC DNA. The reaction was performed at 37°C for 16 h. Digestion products were visualised by 1% agarose gel electrophoresis (24 h, 3 V/cm, 8°C), followed by ethidium bromide staining. Fingerprinting patterns were analyzed in the Image 3.10b gel processing program [55] to generate normalized band position files. Products of vector DNA restriction were masked.

At the first stage of contig assembly, BESs were aligned in Sequencher 4.7 (Gene Codes) to identify BAC clones overlapping at their ends. BAC contigs were constructed in FingerPrinted Contigs, version 8.5.3 [56], with a cut-off 1e-04 and variable tolerance 1.

### Genetic mapping of BAC clones

PCR primer pairs designed for the selected BAC sequences and BESs were used for PCR, based on DNA isolated from parental lines of the *L. angustifolius* mapping population. The population consists of 90 recombinant inbred lines (F_8_) derived from parental lines 83A:476 (D) and P27255 (W) (kindly provided by Dr. Hua’an Yang, Dept. of Agriculture and Food Western Australia). To visualize the number of PCR products obtained, 2 μl of post reaction mixture were subjected to agarose gel electrophoresis. For primer pairs giving single products, amplicons were purified directly from the mixture (QIAquick PCR Purification Kit, Qiagen). In case of primer pairs amplifying two or more products in one reaction, appropriate DNA bands were excised from the gel and recovered (Qiaquick Gel Extraction Kit, Qiagen).

Purified PCR products were sequenced on the ABI PRISM 3130 XL Genetic Analyzer (Applied Biosystems, Hitachi) in order to identify loci with nucleotide sequence polymorphisms. Length polymorphisms of PCR products were visualized by means of agarose gel electrophoresis. Polymorphisms based on nucleotide substitutions were detected by the Cleaved Amplified Polymorphic Sequences (CAPS) approach [45] or derived CAPS (dCAPS) [46]. Restriction sites in CAPS and dCAPS approaches were identified in dCAPS Finder 2.0 [57]. The concentration of agarose gel for electrophoresis in CAPS and dCAPS methods was adjusted accordingly to the size of restriction products, within the range of 2-3%. New markers were localized on the *L. angustifolius* genetic map [17] pursuant to scoring data obtained from the mapping population. The Map Manager QTXb20 program was used for linkage mapping [58]. Graphic illustration of linkage groups was performed in MapChart [59].

### BAC probe preparation for FISH

DNA for molecular probes was isolated from single *Escherichia coli* colonies by means of miniprep method (QIAprep Spin Miniprep Kit, Qiagen), according to Farrar and Donnison [60]. BAC DNA was labeled with digoxygenin-11-dUTP and/or tetramethylrhodamine-5-dUTP (Roche Diagnostics) by nick translation, then subjected to fluorescence *in situ* hybridization (BAC-FISH). Various combinations of reactions were performed, using 2 or 3 BAC probes simultaneously (multiBAC-FISH).

### Chromosome preparation for FISH

BAC-FISH was performed on mitotic metaphase chromosomes. Chromosome squashes were prepared from the root meristems [61] with some modifications for the *L. angustifolius* material [34]. Briefly, after synchronizing and accelerating germination (aeration in tap water at 25°C overnight), the seeds were germinated on moistened filter paper in Petri dishes at 25°C. Seedlings with roots 1.5–2.0 cm long were treated with chilled tap water (2–3°C, 24 h) to accumulate cells at metaphase. Excised roots were fixed in a freshly prepared ethanol and glacial acetic acid mixture (v/v 3:1) and stored at −20°C until use. For cytological preparations, meristematic tissues were digested in the enzyme solution [40% (v/v) pectinase (Sigma, St. Louis, MO), 3% (w/v) cellulose (Sigma), and 1.5% (w/v) cellulase Onozuka R-10 (Serva, Heidelberg, Germany)] for 3–4 h at 37°C. Dissected meristems were squashed on alcohol-cleaned slides in a drop of 60% acetic acid and frozen. Cover slips were removed in liquid nitrogen. The quality of slides was controlled under a phase-contrast microscope (BX41, Olympus).

### Fluorescence *in situ* hybridization (FISH)

FISH was performed according to the protocol published [34]. Preparations were pretreated with RNase (100 μg/ml) in 2× SSC (humid chamber, 37°C, 1 h), washed 3 times in 2× SSC at room temperature (RT), and treated with pepsin (5 μg/ml, 37°C, 12 min). Then the slides were dehydrated in ethanol series (70%, 90%, 100%) and dried (RT). The hybridization mixture (50% deionized formamide, 10% dextran sulfate, 2× SSC, 0.5% SDS, sonicated salmon sperm DNA in 25–100× excess of the probe, 75–200 ng probe per slide) was denatured (90°C, 9 min), applied to the chromosome preparation, and denatured together (78°C, 10 min) using thermal cycler (Twin Tower, PTC-200, MJ Research). Hybridization was carried out at 37°C for 22 h, in a humid chamber. Post-hybridization washes were conducted in 15% deionized formamide in 0.1× SSC at 42°C. Digoxigenated DNA probes were detected with FITC-conjugated antidigoxigenin primary antibodies (Roche Diagnostics). Chromosomes were counterstained with 2 μg/ml DAPI (Sigma) in Vectashield antifade mounting medium (Vector Laboratories, Burlingame, CA). Preparations were examined with the Olympus BX 60 microscope using the Cell_F software, images were captured using a CCD monochromatic camera, and superimposed in Micrografx (Corel) Picture Publisher 8.

## Abbreviations

BAC: Bacterial artificial chromosome; MFLP: Microsatellite fragment length polymorphism; FISH: Fluorescence *in situ* hybridization analysis; GRR: Gene-rich region; AFLP: Amplified fragment length polymorphism; BES: BAC-end sequence; EST: Expressed sequence tag; BLAST: Basic local alignment search tool; CAPS: Cleaved amplified polymorphic sequence; dCAPS: Derived CAPS.

## Competing interests

The authors declare that they have no financial as well as non-financial competing interests.

## Authors’ contributions

MK carried out molecular marker development, genetic mapping, BAC sequence annotation, synteny analysis, participated in contig construction and drafted the manuscript as well as Figures 3 and 4. KW performed BAC-FISH analysis for genetic mapping validation and prepared Figures 1, 2 and 5. AS participated in BAC-FISH studies aimed on verification of physical linkage of particular BACs. SR helped in molecular part of genetic mapping. KM carried out BAC library screening and PCR verification of hybridization results as well as restriction fingerprinting, initial contig assembly and development of 3 molecular markers. ŁP did preliminary BAC-FISH screening of clones to identify single locus BACs. WK performed BAC-end sequence annotation and initial analysis of BAC sequences. BW and BN had a contribution in the general concept of the research scheme and participated in manuscript drafting, especially in sections related to analysis of results and discussion. All authors read and approved the final manuscript.

## Supplementary Material

Additional file 1 The results of BAC-end sequence annotation presented in two separate sections: genes and repetitive elements. Click here for file

Additional file 2 **Detailed marker data including PCR product sizes, sequence identity levels and segregation data for *L. angustifolius *83A:476 (D) and P27255 (W) recombinant inbred lines.** Click here for file
